# Comparison of Primary and Recurrent Urinary Tract Infections in Children

**DOI:** 10.7759/cureus.7019

**Published:** 2020-02-17

**Authors:** Gül Doğan, Hülya İpek

**Affiliations:** 1 Pediatric Surgery, Hitit University Faculty of Medicine, Çorum, TUR

**Keywords:** urinary tract infections, recurrent infection, pediatrics

## Abstract

Aim

We aimed to compare the demographic and ultrasound data regarding first-episode urinary tract infections with recurrent infections in children.

Methods

A total of 509 children aged 0-16 years who were diagnosed to have a urinary tract infection (UTI) as confirmed with positive urinary culture tests were retrospectively investigated. A comparison of baseline parameters, responsible pathogen incidences, and ultrasound findings was made between children who had a single episode of UTI (n=418, 82.1%) with those having second or more recurrent episodes of urinary tract infection (n=91, 17.9%).

Results

The mean age of children with a single episode of urinary tract infection was significantly lower than those who had recurrent urinary tract infection (5.33±4.38 vs. 7.01±4.83 years, p=0.003). Incidences of Escherichia coli and Enterococcus faecalis was significantly higher in patients with recurrent urinary tract infection than those who had single episode (n=315, 75.4% vs. n=80, 87.9%, p=0.009 and n=8, 1.9% vs. n=9, 9.9%, p<0.001, respectively). An abnormal ultrasound was significantly more common in patients with recurrent urinary tract infection than those who had a single episode (n=41, 54.6% vs. n=59, 22.7%). Increased renal parenchymal echogenicity (p=0.002), bladder cystitis (p=0.01) and hydronephrosis (p<0.001) were significantly more common in patients with recurrent urinary tract infection than those who had a single episode of urinary tract infection.

Conclusion

Escherichia coli and Enterococcus faecalis were the most common responsible pathogens in recurrent urinary tract infections. Structural changes, such as hydronephrosis and bladder cystitis, are likely to have an important role in the etiology of children with recurrent urinary tract infection.

## Introduction

Urinary tract infection (UTI) is a worldwide health problem in the pediatric population, with a prevalence of 5.6 -7.0% in young children presenting acutely ill to primary care [1-3]. It has been estimated that 3-7% of girls and 1-2% of boys will be diagnosed with UTI by six years of age, and the disease will recur in 12-30% of these children. Recurrent UTI was defined as having three or more episodes of symptomatic UTI’s within a 12-month-period after the first presentation or two or more episodes within six months. The frequency of recurrent UTI varies depending on the source of data, with the incidence being lower in the primary care departments, whereas higher in the emergency and referral settings [4, 5].

Recurrent UTI may cause chronic symptoms such as abdominal pain, nausea, vomiting, and fatigue. However, the most feared complication of recurrent UTI is renal scarring, which leads to the development of hypertension and chronic renal failure [6]. Although, as reported in many studies, recurrent UTI may be predisposed by underlying structural abnormalities, including bowel dysfunction or vesicoureteral reflux disease, it has been recognized that children without reflux or any other predisposing condition were also at risk for development of recurrent UTI [7, 8]. Recent studies have also demonstrated that antibiotic resistance to common uropathogens is rising due to the increasing rate of extended-spectrum β-lactamase (ESBL) positivity in Escherichia coli (E. coli), Klebsiella pneumonia (K. pneumonia), and other Enterobacteriaceae species and the resistance rates are even higher in children with recurrent infection.

This report presents an analysis of our institutional data from children presenting with first and recurrent episodes of UTI to our outpatient department. We aimed to compare first-episode UTI with recurrent infection regarding demographic and ultrasound characteristics and incidences of responsible uropathogens. We also compare ultrasound findings between first and recurrent episodes to reveal the frequency of structural abnormalities that may be associated with the recurrence of the infection.

## Materials and methods

The study was approved by the local ethics committee (Date 28th June 2019, Number: 2019 -187). The study was conducted in agreement with the Helsinki declaration for studies involving human participants. This retrospective study was conducted in the outpatient department of a tertiary university hospital, and it was made up of patients presented with a first-episode or recurrent episode of a culture-positive urinary tract infection between May 2012 - March 2019. Since this study was a non-interventional archive study, the need for signed informed consent was waived. Medical archive records of a total of 509 children aged 0-16 years who were diagnosed with UTI were included. The diagnose was confirmed by positive urinary culture tests obtained from midstream urine, catheter or suprapubic aspiration which were retrospectively investigated. Patients with urine culture sampling obtained from urinary bag collection were not included. A comparison regarding baseline parameters, responsible pathogen incidences, and ultrasound findings (available in 334 patients) was made between children who had a single episode of UTI (n=418, 82.1%) with those having second or more recurrent episodes of UTI (n=91, 17.9%). Patients’ files were reviewed for baseline information, clinical data, urine culture tests and urinary ultrasound findings. Urinary tract infection was defined as bacterial growth of 105CFU/ML.

Statistical analysis

Statistical analyses were performed using SPSS (Statistical Package for the Social Sciences) version 20.0. (IMB Inc., Armonk, USA). Descriptive statistics were reported as mean ± standard deviation for continuous variables and as frequency and percentage for categorical variables. Categorical variables were compared using the Chi-square test or Fisher exact test where appropriate. Continuous parameters with a normal distribution were compared using independent samples t-test, whereas those not demonstrating normal distribution were compared using the Mann-Whitney test. A p-value of less than 0.05 was considered as statistically significant.

## Results

Baseline characteristics were presented in Table 1. The mean age of children with a single episode of UTI was significantly lower than those who had recurrent UTI (5.33 ± 4.38 vs. 7.01 ± 4.83 years, p=0.003). Gender distribution was similar between the two groups. ESBL positivity was significantly more common in children with recurrent UTI than in those who had a single episode. (n=92, 22.0% vs. n=34, 37.4%, p=0.002).

**Table 1:** Baseline characteristics of patients (n=509) ESBL - extended-spectrum β-lactamase; SD - standard deviation * Presence of at least one positive culture with the pathogen microorganism

Variable	Primary (n=418, 82.1%)	Recurrent (n=91, 17.9%)	p-values
Age (years) mean (SD)	5.33 ± 4.38	7.01 ± 4.83	0.003
Females	287 (68.7%)	69 (75.8%)	0.17
ESBL positive	92 (22.0%)	34 (37.4%)	0.002
Pathogen microorganisms*			
Escherichia coli	315 (75.4%)	80 (87.9%)	0.009
Klebsiella pneumonia	52 (12.4%)	12 (13.2%)	0.86
Enterococcus faecalis	8 (1.9%)	9 (9.9%)	<0.001
Proteus mirabilis	9 (2.2%)	8 (8.8%)	0.005
Enterococcus faecium	8 (1.9%)	3 (3.3%)	0.41
Klebsiella oxytoca	6 (1.4%)	0 (0%)	0.59
Morganella morganii	3 (0.7%)	3 (3.3%)	0.07
Pseudomonas aeruginosa	2 (0.5%)	3 (3.3%)	0.04
Staphylococcus aureus	4 (1.0%)	0 (0%)	0.99
Staphylococcus haemolyticus	3 (0.7%)	1 (1.1%)	0.54
Staphylococcus hominis	2 (0.5%)	1 (1.1%)	0.44
Enterobacter aerogenes	1 (0.2%)	2 (2.2%)	0.08
Enterobacter amnigenus	2 (0.5%)	0 (0%)	0.99
Streptococcus agalactiae	1 (0.2%)	1 (1.1%)	0.32
Acinetobacter baumannii	1 (0.2%)	0 (0%)	0.99

In both groups, Escherichia coli was the most common pathogen, and Klebsiella pneumonia was the second most common pathogen in urine cultures. Incidences of Escherichia coli and Enterococcus faecalis were significantly higher in patients with recurrent UTI than those who had single episode (n=315, 75.4% vs. n=80, 87.9%, p=0.009 and n=8, 1.9% vs. n=9, 9.9%, p<0.001, respectively).

Ultrasound data was available for 259 patients (61.9%) with a single episode and for 75 patients (82.4%) with recurrent UTI (p<0.001). An abnormal ultrasound was significantly more common in patients with recurrent UTI than those who had a single episode (n=41, 54.6% vs. n=59, 22.7%). Increased renal parenchymal echogenicity (p=0.002), bladder cystitis (p=0.01), and hydronephrosis (p<0.001) were significantly more common in patients with recurrent UTI than those who had a single episode of UTI (Table 2).

**Table 2:** Ultrasound findings (n=334, available cases)

Variable	Primary (n=259, 77.5%)	Recurrent (n=75, 22.5%)	p-value
Abnormal	59 (22.7%)	41 (54.6%)	<0.001
Increased renal parenchymal echogenicity	2 (0.8%)	5 (6.7%)	0.002
Bladder cystitis	18 (6.9%)	12 (16.0%)	0.01
<3 mm hyperechoic foci within renal calyces	10 (3.9%)	3 (4.0%)	0.96
Hydronephrosis	29 (11.2%)	21 (28.0%)	<0.001
Persistent ultrasound findings (n=93)	36 (66.7%)	19 (48.7%)	0.08

## Discussion

The results of our study showed that Escherichia coli was the most common responsible pathogen in both primary and recurrent urinary tract infections whereas Enterococcus faecalis was significantly higher in children with recurrent urinary tract infection than those having their first episode. We think that these findings are in line with those reported in a recent review study, which found a significant association with bowel dysfunction and recurrent urinary tract infection in children [3]. The authors of the study reported that 35% of children with bladder-bowel dysfunction and 51% of children with concomitant bladder-bowel dysfunction and vesicoureteral reflux had recurrent urinary tract infection.

Our findings are supported by a large-scale study where a total of 1,045 bacteria identified from urine culture samples obtained from pediatric patients. In that study, Escherichia coli was found in 60.3% of the samples, and it was followed by Enterococcus faecalis (22.4%) and Klebsiella spp. (6.5%) [9]. Similarly, another study reported that Escherichia coli was the most common responsible pathogen (51.5%), and it was followed by Klebsiella spp. (16.8%) and Enterococcus spp. (9.9%) in 202 children aged between two months to 18 years [10].

Our data revealed that about 80% of patients with recurrent episodes had an ultrasound imaging, and more than half of them had an abnormal ultrasound finding. Hydronephrosis was the most common ultrasound finding, whereas bladder cystitis, which is of pathophysiologic importance, was found significantly more common in patients with the recurrent episode. In line with our findings, Yilmaz et al. [11] reported that recurrent urinary tract infections were significantly more common in patients with renal scarring and structural abnormalities. The authors reported that the rate of recurrent infection was as high as 55%, and the presence of an ultrasound abnormality was 41.2% in children older than five years.

Our study had several limitations, including the retrospective design, and lack of culture-antibiogram susceptibility data. Although ultrasound data was available in the majority of patients with recurrent episodes, one-third of the patients with single episode did not have ultrasound examinations, and thus an unequivocal comparison of ultrasound data between two groups was not possible. 

## Conclusions

We saw that Escherichia coli and Enterococcus faecalis were the most common responsible pathogens in recurrent urinary tract infections. Structural changes, such as hydronephrosis and bladder cystitis, are likely to have an important role in the etiology of children with recurrent urinary tract infections.
